# Selection of photosynthetic traits by turbulent mixing governs formation of cyanobacterial blooms in shallow eutrophic lakes

**DOI:** 10.1093/ismejo/wrae021

**Published:** 2024-02-02

**Authors:** Huaming Wu, Xingqiang Wu, Lorenzo Rovelli, Andreas Lorke

**Affiliations:** Institute for Environmental Sciences, University of Kaiserslautern-Landau (RPTU), Landau 76829, Germany; Key Laboratory of Algal Biology of Chinese Academy of Sciences, Institute of Hydrobiology, Chinese Academy of Sciences, Wuhan 430072, China; Institute for Environmental Sciences, University of Kaiserslautern-Landau (RPTU), Landau 76829, Germany; Now at the Department of Ecology, Federal Institute of Hydrology (BfG), Koblenz 56068, Germany; Institute for Environmental Sciences, University of Kaiserslautern-Landau (RPTU), Landau 76829, Germany

**Keywords:** aquatic ecosystems, cyanobacteria-environment interactions, surface scum, intraspecific variation, population dynamics, photosynthetic capacity

## Abstract

Prediction of the complex cyanobacteria-environment interactions is vital for understanding harmful bloom formation. Most previous studies on these interactions considered specific properties of cyanobacterial cells as representative for the entire population (e.g. growth rate, mortality, and photosynthetic capacity (*P_max_*)), and assumed that they remained spatiotemporally unchanged. Although, at the population level, the alteration of such traits can be driven by intraspecific competition, little is known about how traits and their plasticity change in response to environmental conditions and affect the bloom formation. Here we test the hypothesis that intraspecific variations in *P_max_* of cyanobacteria (*Microcystis* spp*.*) play an important role in its population dynamics. We coupled a one-dimensional hydrodynamic model with a trait-based phytoplankton model to simulate the effects of physical drivers (turbulence and turbidity) on the *P_max_* of *Microcystis* populations for a range of dynamic conditions typical for shallow eutrophic lakes. Our results revealed that turbulence acts as a directional selective driver for changes in *P_max_*. Depending on the intensity of daily-periodic turbulence, representing wind-driven mixing, a shift in population-averaged phenotypes occurred toward either low *P_max_*, allowing the population to capture additional light in the upper layers, or high *P_max_*, enhancing the efficiency of light utilization. Moreover, we observed that a high intraspecific diversity in *P_max_* accelerated the formation of surface scum by up to more than four times compared to a lower diversity. This study offers insights into mechanisms by which cyanobacteria populations respond to turbulence and underscores the significance of intraspecific variations in cyanobacterial bloom formation.

**Highlights:**

1) Numerical simulations of *Microcystis* population dynamics including intraspecific variations.
2) Moderate turbulence can induce a stable reduction in the photosynthetic capacity of the population.
3) High diversity in photosynthetic capacity accelerates scum formation.

## Introduction

Harmful cyanobacterial blooms occur more frequently and more intensely at global scale as the environment is increasingly impacted by anthropogenic eutrophication, pollution, and extreme climate [1, 2]. Although various studies have focused on how abiotic and biotic environmental factors affect cyanobacterial population dynamics [3–6], they implicitly assumed that the variability of observed traits is independent of population dynamics and environmental stressors, such that a set of averaged trait properties adequately represents the population, regardless of the time or location. However, there is growing evidence that this simplified assumption may under-represent the importance of variation between individuals and community structure in population ecology [7-9]. As intraspecific variations have also been observed in cyanobacteria [10–13], their ability for bloom formation under diverse environmental conditions is potentially affected by the coexistence or competition of different traits.

Under the influence of environmental stressors, intraspecific variation is fundamental for driving intraspecific competition and thus shaping the population response. Selection favors traits that enhance individual fitness, leading to their increased prevalence under selective pressures and subsequently reshaping the distribution of traits. This process typically occurs over demographic timescales [14, 15]. Cyanobacteria, renowned for their rapid growth, are capable of doubling their population size within 1–2 days under ideal conditions [16]. This implies that cyanobacteria may have a high potential for selection, by which cyanobacterial populations accumulate favorable traits to cope with contrasting environments.

Photosynthetic capacity is crucial among multiple traits of cyanobacteria that drive the occurrence of surface blooms [17, 18]. Through the process of photosynthesis, cyanobacteria are converting sunlight into energy, which fuels cell division and metabolic processes, including the carbon-reserve metabolism [19]. The carbon-reserve metabolism is associated with buoyancy regulation and affects the vertical position of cyanobacteria in the water [20]. The mass density of cyanobacterial cells is modulated by the rate of carbohydrates produced through photosynthesis or consumed via respiration within the cells. This mechanism enables cyanobacteria to gain access to well-lit surface waters by consuming intracellular ballast, and access to nutrients at larger depths after storing sufficient carbohydrates. This mechanism has been extendedly used in predictive models for bloom dynamics [21, 22].

Given the considerable intraspecific variation in photosynthetic capacity of cyanobacteria, which can range over one order of magnitude [23, 24], it can be expected that traits with differing photosynthetic capacities migrate along different trajectories. This can lead to competition for light among traits and to interactions, e.g. by mutual shading, resulting in distinct life histories. Abiotic factors that regulate cyanobacterial bloom dynamics in lakes, such as turbulence and turbidity, may interact with the intraspecific light competition. High turbidity, for example, can diminish the availability of light in lakes, thereby directly affecting the ambient light prevailing traits, i.e. their light niches. Turbulence, in contrast, can control the vertical distribution of cyanobacteria, potentially either confining or relaxing the boundaries between the light niches utilized by traits with different photosynthetic capacities. A mechanistic understanding of such complex interactions between biotic and abiotic processes and the resulting cyanobacterial population dynamics is still lacking, and so is our understanding of their environmental relevance, e.g. for bloom formation.

In the natural water bodies, these interactions are affected and potentially masked by synoptic and seasonal variations in the physical forcing. Therefore, numerical modeling of the trait dynamics under idealized and simplified environmental conditions (e.g. periodic light and wind forcing) are a more appropriate approach for analyzing such complex interactions.

In this study, we implemented a simplified one-dimensional hydrodynamic model with a trait-based phytoplankton model to examine how turbulence and turbidity affect the photosynthetic capacity of cyanobacterial populations through trait selection and analyze the resulting changes in population composition during bloom formation. The model was used to simulate the growth and vertical distribution of colony-forming *Microcystis* populations with varying ranges of different photosynthetic capacities under commonly occurring turbulence and turbidity levels in lakes. We hypothesize that the photosynthetic capacity of a *Microcystis* population can be substantially altered by turbulence and turbidity and that this selection plays an important role during surface bloom formation. This study is thus expected to be instrumental in advancing our understanding of the cyanobacteria-environment interactions and their role during bloom formation.

## Materials and methods

### Model description

#### General description

Our model consists of three components: (i) a one-dimensional hydrodynamic model, (ii) an ensemble-averaged transport model for simulating the trait-specific vertical distribution dynamics of cell number concentration, colony size, and cell-tissue density, and (iii) an ecological model describing cell and colony photosynthesis and growth. The maximum photosynthetic rate (maximum rate of photosynthesis at optimal light intensity, normalized by carbon content, *P_max_* in s^−1^, [25]) was used to characterize the different photosynthetic capacity of *Microcystis* traits and was assumed to vary within different ranges for different initial population. We discretized the range of *P_max_* observed for *Microcystis* populations [26–29] into 10 evenly spaced subranges, resulting in 10 trait groups (g_1_-g_10_), with increasing *P_max_*. The population dynamics was simulated by simultaneously simulating the vertical distribution dynamics of the ten trait groups. The vertical distribution of each trait groups was obtained by solving the extended Langevin–Fokker–Planck equation (see below). While solving this equation for cell density (growth and loss processes), colony size, light-mediated changes in cell tissue density, and vertical colony migration (floating or sinking) velocity, as well as vertical turbulent mixing are considered as dynamic parameters. External environmental conditions include diel variations in turbulent diffusivity and light intensity. The latter interacts with the vertical distribution of the *Microcystis* population through self-shading, which is considered in addition to different background light attenuation coefficients, representing different water turbidity. As such, different trait groups can interact with each other by mutual shading. The models are described in detail below, while the main parameters, their numerical values and reference for parameter selection are summarized in the Supporting Information (Table S1).

The dynamics of *Microcystis* populations were simulated under simplified conditions representing an idealized shallow eutrophic lake, where we disregarded the effects of temporally and vertically varying water temperature and nutrient limitation. We assumed that *Microcystis* growth depends solely on irradiance, which varies with a diel cycle, and that a fixed *P_max_* applies to each trait group (see Supporting Information Text S1 for more details).

#### Distribution dynamics of *Microcystis* populations

The vertical dynamics of the cell density (*C_i_*) of the trait group *i* (*i* = 1 … 10) was modeled by the extended Langevin–Fokker–Planck equation [30, 31], as follows:


(1) \begin{equation*} \frac{\partial{c}_i}{\partial_t}=-\left(\frac{\partial \rho }{\partial_t}\right)\;\frac{\partial{c}_i}{\partial_{\rho }}-\left(\frac{\partial d}{\partial_t}\right)\;\frac{\partial{c}_i}{\partial_d}-\left(\frac{\partial z}{\partial_t}\right)\;\frac{\partial{c}_i}{\partial_z}+\frac{\partial }{\partial_z}\left({D}_z\frac{\partial{c}_i}{\partial_z}\right)+\left(r-l\right){C}_i \end{equation*}

Eq. 1 is a one-dimensional advection–diffusion equation commonly used to simulate the vertical migration of cyanobacteria under turbulence [31–33]. *C*_i_ (*z*, *ρ*, *d*, *t*) is the concentration of cells aggregated to colonies of size *d* (μm), at depth *z* (m) and time *t* (s), and with a cell-tissue density *ρ* (kg m^−3^). By this definition, the distribution varies along the cell-tissue density coordinate *ρ*, colony size coordinate *d* as well as along physical depth coordinate *z*, and time *t*. The index *i* stands for different trait groups (g_1_ –g_10_) of the population. The first three terms on the right-hand side of Eq. 1 are advective terms in the *ρ*, *d*, and the vertical coordinate (*z*), respectively. Herein, *∂z/∂t*, *∂ρ/∂t*, and *∂d/∂t* denote the rates of change of depth, cell density, and colony size of the respective trait groups. The fourth term describes vertical transport by turbulent diffusion with *D_z_* (m^2^ s^−1^) denoting the turbulent diffusivity at depth *z*. The last term describes growth and loss processes with *r* and *l* being growth and loss (mortality) rates of *Microcystis*. We define the direction of *z* is positive downward.

#### Photosynthesis model of *Microcystis*

A classical *P*-*I* relationship was used to relate the photosynthetic rate (*P*, s^−1^) to irradiance (*I*) as follows [34, 35]:


(2) \begin{equation*} P=\frac{I}{\left(a{I}^2+ bI+c\right)} \end{equation*}

where *a*, *b*, and *c* are related to characteristic photosynthesis parameters (i.e. the initial slope (*S*), the maximum photosynthetic rate (*P_max_*) and the optimal light intensity (*I_opt_*) can be expressed in terms of the parameters as: *S* = 1/*c*; *I_opt_* = (*c*/*a*)^1/2^; *P_max_* = 1/(*b* + 2*(*ac*)^1/2^) [34]). In this study, we assumed constant values for *I_opt_* (~277.5 μmol photons m^−2^ s^−1^, [36, 37]) and *S* (2 × 10^−7^ (μmol photons)^−1^ m^2^, [27]). Especially the latter has been found to be almost constant from March to October (the period of a *Microcystis* bloom) in lake Kasumigaura [27].

#### Growth, loss, and cell tissue density of *Microcystis*

The rate of carbon fixation by photosynthesis (*P* in Eq. 2) is allocated to cell growth (*r*), the rate of change in ballast (characterized by the rate of change in cell tissue density, *∂ρ/∂t*), and the respiration rate (*R)*, following as [36, 38]:


(3) \begin{equation*} r=\left\{\begin{array}{@{}c}{g}_{max}, if\ P-R\ge{g}_{max}\\P-R, if\ {g}_{max}> P-R\ge 0\\0, if\ P-R < 0 \end{array}\right. \end{equation*}

(4) \begin{equation*} \frac{\partial \rho }{\partial t}=\left\{\begin{array}{@{}c}{m}_{cell}\cdot \left(P-R-{g}_{max}\right)\cdot \frac{B_g}{V_{cell}}, if\ P-R\ge{g}_{max}\\ 0, if\ {g}_{max} > P-R\ge 0\\ \left({f}_1\cdot{\rho}_i + {f}_2\right)/60, if\ P-R < 0 \end{array}\right. \end{equation*}

where *g_max_* is the maximum carbon uptake rate for growth (set to a constant value of 5.5 × 10^−6^ s^−1^, corresponding to the growth rate of 0.48 d^−1^), as it is independent of *P_max_* [24]. The respiration rate (*R*) was assumed to remain constant at 0.55 × 10^−6^ s^−1^ [25]. *B_g_* represents the mass of carbohydrate (glycogen) ballast produced per gram of assimilated carbon, with a value of 2.38 and *V_cell_* is the volume a single *Microcystis* cell (taken to be 67 × 10^−18^ m^3^), and *m_cell_* is the amount of carbon contained in each cell (14 × 10^−15^ kg) [25]. The loss rate of *Microcystis* (*l*) was assumed to be constant (0.1 d^−1^, [33]). *f_1_* (min^−1^) is the slope of the curve of density change, *f_2_* is the theoretical rate (kg m^−3^ min^−1^) of density change with no carbohydrate storage in the cells and *ρ_i_* is the initial density (kg m^−3^).

#### Colony size, migration velocity of *Microcystis*, and external environmental conditions

The interactions between environmental conditions and colony size dynamics are complex and involve cell division, cell adhesion, and colony disaggregation. For simplicity, we neglect cell adhesion as colonies formed through adhesion are more readily disaggregated [39]. Hence, colonies consist of cells of a single genotype and identical photosynthetic capacity in this study. The colony size was modulated by the growth and mortality (loss) of *Microcystis* cells and was constrained by an upper limit (420 μm), which corresponds to the largest stable colony size under turbulent conditions [40]. Colonies grow and shrink in size, depending on whether net grows (*r* - *l*) is positive, or negative, respectively. The rate of change of colony diameter (∂*d*/∂*t*) was approximated as a function of net cell growth (*r* - *l*) following [22]:


(5) \begin{equation*} \frac{\partial d}{\partial t}\approx{d}_{t-1}\left({2}^{\frac{\triangle t\cdot \left(r-l\right)}{\ln (2)}}-1\right)/\triangle t \end{equation*}

where *d_t-1_* is the colony size at the previous time step (*t*-1) and *Δt* is the time step in the discretized numerical solution. This equation is derived from the mechanism of colony formation, where the size of the colony increases through cell division [39]. Thus, colony size is indirectly linked to photosynthetic capacity.

The vertical velocity of *Microcystis* follows Stokes’ law (Text S2). Due to varying drivers (e.g. wind shear versus convection) and the influence of density stratification, there is no universal profile for the vertical distribution of turbulent diffusivity in lakes. For simplicity, we adopted the empirical vertical turbulent diffusivity (*D_z_*) profile from a previous study [41], wherein the turbulent diffusivity is fixed throughout an upper layer of the lake (~10% of water depth), and declines with depth following a parabolic profile below this layer (see Text S3). In the following, we used the maximum *D_z_* (*D_z,max_*) within each profile for referring to the corresponding *D_z_* profiles. The irradiance profiles were simulated by Lambert–Beer’s law, considering the self-shading of the *Microcystis* population and a constant background turbidity that was varied in our simulations (Text S3).

### Simulation setting

In our simulations, the water depth was fixed to 3 m with a resolution of the vertical discretization of 0.05 m, the cell tissue density ranged from 996 to 1130 kg m^−3^ with a resolution of 3.35 kg m^−3^ [31], and the colony size ranged from 10 to 420 μm with resolution of 10 μm [42]. No-flux conditions are applied at the boundaries of *z*, *ρ*, and *d* axes.

The initial cell density for all trait groups of the *Microcystis* population was uniform throughout the water column, with an initial colony size of 50 μm. The uniform vertical distribution and consistent colony size are widely used initial conditions in former studies [31, 33]. Given the unknown trait distribution in nature, we also used an initially uniform distribution to allow different trait groups to compete equally. The chosen initial colony size and cell density (2 × 10^4^ cells mL^−1^) correspond to conditions observed in the early phase (~April) of *Microcystis* blooms in lake Taihu [43]. The initial density of colonies was set to 998 kg m^−3^ (neutrally buoyant).

All simulations started at 6:00 and were performed for 180 consecutive days. Depending on the turbulent eddy diffusion coefficient, the temporal resolution of the simulations was varied from 1.2 to 120 s to ensure that the time step met the stability condition for Eq. 1 [44].

We used an iterative algorithm at each time step (time-stepping solver) to solve the above equations and to obtain the dynamic distributions of all trait groups of *Microcystis* colonies throughout the water column. The solver was implemented through a self-written computer program in MATLAB 2022b, where the first and second derivatives were approximated using a combination of forward and central differences.

**Figure 1:**
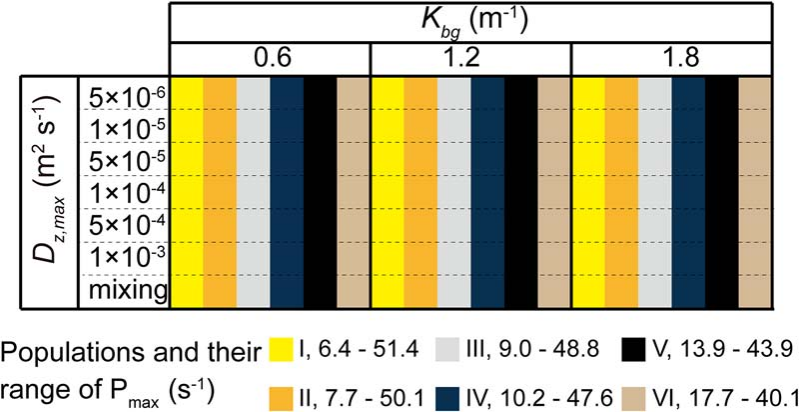
Experimental design of the numerical simulations, which were conducted for all combinations of different initial *Microcystis* populations (I-VI with varying ranges of photosynthetic capacity, *P_max_*, marked by different color), different turbulent diffusivities (*D_z,max_*) and different turbidity (*K_bg_*) conditions.

We used six *Microcystis* populations with initially varying ranges of *P_max_* (population I – VI, Fig. 1). The arithmetic mean *P_max_* of each population was 28.9 × 10^−6^ s^−1^. Each population is composed of ten trait groups (g_1_-g_10_), which differ among different populations. We simulated the dynamics of the populations under seven different turbulence conditions and with three different background extinction coefficients, resulting in a total of 126 simulations (6 × 7 × 3, Fig. 1). Except for the complete mixing condition, the turbulent conditions used in this study followed a diel pattern, with a constant diffusivity of *D_z,max_* = 10^−6^ m^2^ s^−1^ applied during nighttime (18:00–6:00), while higher constant diffusivities during daytime (6:00–18:00) were varied between *D_z,max_* = 5 × 10^−6^, 1 × 10^−5^, 5 × 10^−5^, 1 × 10^−4^, 5 × 10^−4^, and 1 × 10^−3^ m^2^ s^−1^ (turbulent diffusivity change suddenly in the morning and evening). Under complete mixing conditions, we assumed uniform vertical distribution of *Microcystis* cell density throughout the water column and excluded diel patterns, thus focusing solely on the growth and loss of *Microcystis*. The range of turbulent diffusivities used in this study correspond to the observed range in natural lakes and the complete mixing represent strong turbulence such as storm conditions [45]. The respective ranges of *P_max_* for different populations, turbulence conditions (*D_z,max_*), and light extinction coefficients (*K_bg_*) used in the simulations are summarized in Fig. 1.

### Data processing

The mean vertical positions of trait groups were described by the centroids (*z_c_*) of their vertical distributions:


(6) \begin{equation*} {z}_c=\frac{\sum_{j=1}^m{C}_j\cdot{z}_j}{\sum_{j=1}^m{C}_j} \end{equation*}

where *z_c_* is the centroid of the population/trait group, *m* is the number of vertical layers (*m* = 60), *C_j_* is the cell density in the *j^th^* layer, and *z_j_* is the depth of the *j^th^* layer.

The population-averaged photosynthetic capacity of *Microcystis* ($\overline{P}$*_max_*) was characterized by the average value of all trait groups:


(7) \begin{equation*} \overline{P_{max}}=\frac{\sum_{i=1}^n\overline{C_i}\cdot{P}_{\max, i}}{\sum_{i=1}^n\overline{C_i}} \end{equation*}

where *n* is the number of trait groups in each population (*n* = 10), $\overline{C}_{i}$ and *P_max,i_* are the mean (vertically averaged) cell density and the maximum photosynthetic rate of each trait group (g*_i_*).

The distribution of irradiance in the water column was characterized by the euphotic depth, which was calculated as the depth at which the light intensity decreased to 1% of its maximum value at the water surface.

The relative cell density of trait groups (*RC*) was used as a proxy for the distribution of traits with different photosynthetic capacity within the populations. This was determined by calculating the ratios of trait group cell density to total population cell density.

We established specific thresholds to differentiate different levels of blooms and surface scum for the *Microcystis* population (see Supporting Information Text S4 for more details).

## Results

### Effect of turbulence on population composition and photosynthetic capacity

**Figure 2:**
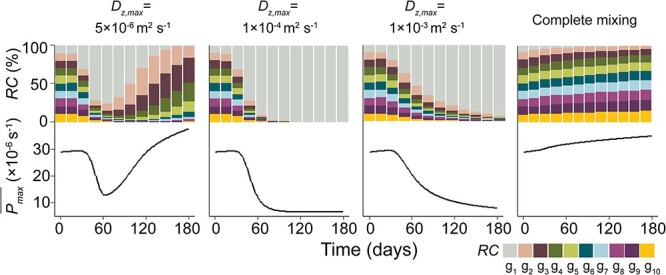
Population dynamics of *Microcystis* (population I) for four different turbulence conditions (different columns: *D_z,max_* = 5 × 10^−6^ m^2^ s^−1^; *D_z,max_* = 1 × 10^−4^ m^2^ s^−1^; *D_z,max_* = 1 × 10^−3^ m^2^ s^−1^; complete mixing). The upper panels show the relative cell density (*RC*) of the 10 different trait groups (g_1_ – g_10_), while the lower panels show the population-averaged photosynthetic capacity of the *Microcystis* population ($\overline{P}$*_max_***,** solid lines). The cell densities (*RC*) are shown as staggered area charts. Group g_1_ has the minimum *P_max_*, while g_10_ has maximum *P_max_*. The turbidity conditions were *K_bg_* = 0.6 m^−1^.

We observed an exponential growth of the population during the initial phase, which levelled off after 90–180 days when the cell densities reached constant maximal values for all simulated turbulence and turbidity conditions (Fig. S1, see online supplementary material for a colour version of this figure). Similarly, the mean population depth (i.e. the depth of its centroid) showed an initial decrease followed by an increase and eventual stabilization in the upper part of the water column under all turbulence conditions, except for the complete mixing (Fig. S2, see online supplementary material for a colour version of this figure). The hourly time series of the centroid depth of the population exhibits a diel pattern, with the centroid increasing during daytime and decreasing during nighttime (Fig. S3, see online supplementary material for a colour version of this figure). However, the population composition and the population-averaged *P_max_* ($\overline{P}$*_max_*) responded differently to turbulence and turbidity conditions (Figs S4 and S5, see online supplementary material for a colour version of this figure). We identified four representative turbulence-dependent patterns in population composition dynamics, which we exemplarily describe for the lowest turbidity (0.6 m^−1^, Fig. 2).

Under weak turbulence (*D_z,max_* = 5 × 10^−6^ m^2^ s^−1^), the relative cell density (*RC*) of the trait groups was similar during the initial 30-day period, with a nearly constant $\overline{P}$*_max_* (Fig. 2). The centroid of the *Microcystis* trait group with the lowest photosynthetic rate (g_1_) moved rapidly to the water surface (Fig. S6, see online supplementary material for a colour version of this figure). This is primarily due to its higher floating velocity (Fig. S7A, see online supplementary material for a colour version of this figure), which results from a rapid decrease in mass-density (Fig. S7C, see online supplementary material for a colour version of this figure), compared to the other groups. Consequently, its cell density increased rapidly between 30 and 60 days (Fig. S6A, see online supplementary material for a colour version of this figure), leading to an increase in the *RC* of g_1_, while the $\overline{P}$*_max_* decreased (Fig. 2). In contrast to g_1_, trait groups g_2_ –g_10_ showed opposing migration behavior during the first 30 days, with increasing depths of the centroids of their distributions (Fig. S6B, see online supplementary material for a colour version of this figure) due to high mass-densities (Fig. S7C, see online supplementary material for a colour version of this figure). However, during 30–180 days, their centroid depths decreased and gradually approached the water surface (Fig. S6, see online supplementary material for a colour version of this figure) and their cell densities increased (Fig. S6A, see online supplementary material for a colour version of this figure). This change is a consequence of their decreasing mass-densities (Fig. S7C, see online supplementary material for a colour version of this figure), likely due to increasing self-shading (decreasing euphotic depth, Fig. S6B, see online supplementary material for a colour version of this figure). At Day 60, the cell density of the group *g*_1_ started to decrease, while its centroid remained at the water surface for a period of around 40 days before continuously increased to larger depths after Day 100 (Fig. S6A, see online supplementary material for a colour version of this figure). The decline of g_1_ is likely attributed to the competitive advantage of g_2_ –g_10_ (with higher *P_max_*) over g_1_ at the water surface. In addition, there was an increase in the *RC* of the trait groups g_2_ –g_10_ after 60 days, leading to a reversal of the decline and a continuous increase of the $\overline{P}$*_max_* (Fig. 2). At Day 180, the population was dominated by the trait groups g_2_ –g_6_.

Under moderate turbulence (e.g. *D_z,max_* = 1 × 10^−4^ m^2^ s^−1^), the centroid of trait group g_1_ initially decreased and stabilized at the water surface after 20 days (Fig. S6B, see online supplementary material for a colour version of this figure). As time progressed, the cell density of g_1_ increased continuously, while that of the remaining trait groups (g_2_ –g_10_) initially increased, followed by a subsequent decrease (Fig. S6A, see online supplementary material for a colour version of this figure). Simultaneously, an increasing *RC* of g_1_ within the *Microcystis* population and a reduction of the $\overline{P}$*_max_* was observed (Fig. 2). The trait groups g_2_ –g_10_, unlike under weak turbulence, did not accumulate at the water surface, which can be explained by the higher turbulent dispersion as well as their low floating velocities (~ 0 μm s^−1^, Fig. S7A, see online supplementary material for a colour version of this figure). The latter is the results of their decreasing colony size (Fig. S7B, see online supplementary material for a colour version of this figure), likely caused by their declining growth (cf. eq. 5). Consequently, the decrease in the $\overline{P}$*_max_* did not reverse under moderate turbulence. After 90 days, the trait group *g*_1_ had become the dominant trait group the *Microcystis* population, with a *RC* exceeding 99.9% (Fig. 2). This indicates that moderate turbulence could result in a stable population dominated by almost a single trait of low *P_max_*.

Under strong turbulence (e.g. *D_z,max_* = 1 × 10^−3^ m^2^ s^−1^), the centroid of trait group *g*_1_ did not reach the water surface, but it still remained at shallower depths than those of trait groups g_2_ –g_10_ (Fig. S6B, see online supplementary material for a colour version of this figure). After 60 days, there was a significant increase in the cell density and *RC* of trait group g_1_ (Fig. S6A, see online supplementary material for a colour version of this figure and Fig. 2). This resulted in a continued decrease in the $\overline{P}$*_max_*, similar to what was observed for moderate turbulence (Fig. 2). However, unlike the population composition observed under moderate turbulence, the *RC* of trait groups g_2_ –g_10_ still exceeded ~8% under high turbulent conditions at the end of the simulation (Fig. 2).

Complete mixing homogenized the population throughout the water column, leading to a location of the centroid of all trait groups at mid depth (Fig. S6B, see online supplementary material for a colour version of this figure). The cell density of each trait group was positively correlated with its corresponding *P_max_*: Over the entire simulation period of 180 days, *Microcystis* trait groups with high *P_max_* gradually developed higher cell densities and *RC* (Fig. S6A, see online supplementary material for a colour version of this figure and Fig. 2), suggesting that trait groups with higher photosynthetic rates were more competitive and had slightly higher growth rates under completely mixed conditions. This was associated with a simultaneous increase in the $\overline{P}$*_max_* of the entire *Microcystis* populations (Fig. 2).

### Effect of turbidity on population composition and photosynthetic capacity

**Figure 3:**
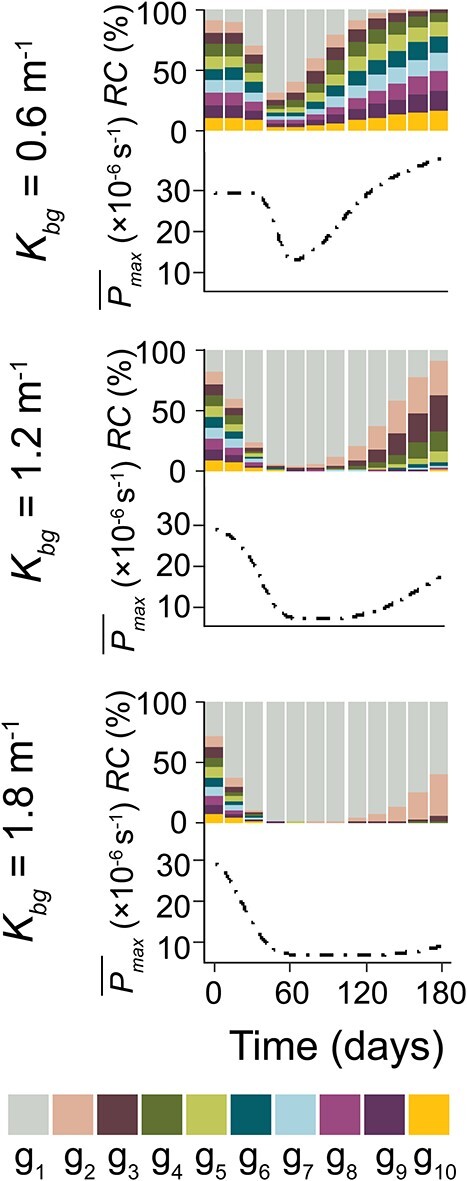
The effect of turbidity on the population dynamics of *Microcystis* (population I) under weak turbulence (*D_z,max_* = 5 × 10^−6^ m^2^ s^−1^) (see different rows: *K_bg_* = 0.6 m^−1^; *K_bg_* = 1.2 m^−1^; *K_bg_* = 1.8 m^−1^): The upper panel in each row show the relative cell density (*RC*) of the 10 different trait groups (g_1_ – g_10_) as staggered area charts, while the lower panel in each row show the population-averaged photosynthetic capacity of the *Microcystis* ($\overline{P}$*_max_*, solid,). Group g_1_ has the minimum *P_max_*, while g_10_ has maximum *P_max_*.

The population composition dynamics of *Microcystis* generally followed the typical pattern for different turbulence regimes (as we present in section: Effect of turbulence on population composition and photosynthetic capacity) across the range of tested turbidity (Fig. S4, see online supplementary material for a colour version of this figure). Although the pattern of the $\overline{P}$*_max_* was primarily influenced by turbulence, we observed that turbidity affected time when the $\overline{P}$*_max_* began to decline and reverse under low turbulence conditions. Here we exemplarily presented these results for the lowest turbulence (*D_z,max_* = 5 × 10^−6^ m^2^ s^−1^, Fig. 3).

Under different turbidities, the $\overline{P}$*_max_* consistently exhibited a pattern of initial stability, followed by a decline, and eventual increase (Fig. 3). All trait groups initially exhibited a similar growth trend (Fig. S8A, see online supplementary material for a colour version of this figure), during which $\overline{P}$*_max_* remained relatively constant. However, higher turbidity accelerated the decline of $\overline{P}$*_max_* compared to lower turbidity (Fig. 3). In addition. as turbidity increased, the time at which $\overline{P}$*_max_* started to change (defined as a change from the initial $\overline{P}$*_max_* of more than 5%) decreased from 40 to 12 days and 7 days for background extinction coefficients (*K_bg_*) of 0.6 , 1.2, and 1.8 m^−1^ (Fig. S9, see online supplementary material for a colour version of this figure).

Turbidity also affected the reversal of the declining $\overline{P}$*_max_* under low turbulence. High turbidity (1.8 m^−1^) delayed the occurrence of the reversal (the time at which $\overline{P}$*_max_* undergoes a change in direction from decreasing to increasing) compared to low turbidity (0.6 m^−1^) by a factor of 2.5 (Fig. 3). This can be explained by the observation that low turbidity led to larger colony size of g_2_-g_10_ and therefore their higher floatation velocity (Fig. S10, see online supplementary material for a colour version of this figure), facilitating their faster upward migrations than under high turbidity (Fig. S8B, see online supplementary material for a colour version of this figure). As a result, the trait distribution of the population at the end of the simulation (180 days) differed for different turbidities (Fig. 3). Under low turbidites, the population was dominated by traits with higher photosynthetic capacity (g_3_ –g_10_), while under high turbidity, the population was dominated by traits with low photosynthetic capacity (g_1_ and g_2_).

### Effect of intraspecific variation on bloom and scum formation

We simulated the processes of bloom and scum formation of *Microcystis* population with varying initial ranges of photosynthetic capacity (Population I - Population VI) under different turbulence and turbidity. The simulated cell densities showed good consistency with those in the supplementary simulations performed with different initial conditions for the vertical distribution of the colonies and for initial colony size (Fig. S11, see online supplementary material for a colour version of this figure).

**Figure 4:**
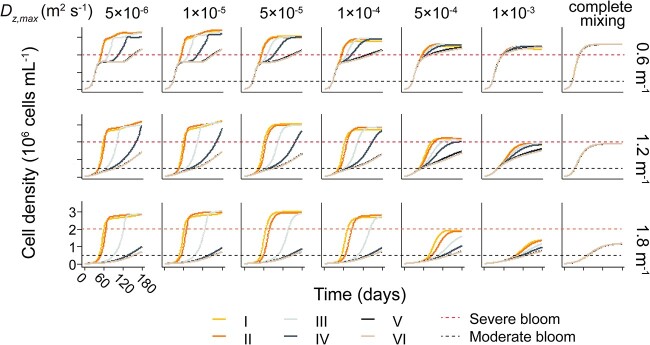
Time series of mean cell density of *Microcystis* populations with different spectra of *P_max_* (population I to IV, see legend) under different turbulence (columns) and turbidity (characterized by *K_bg_* in m^−1^ for the different rows).

**Figure 5:**
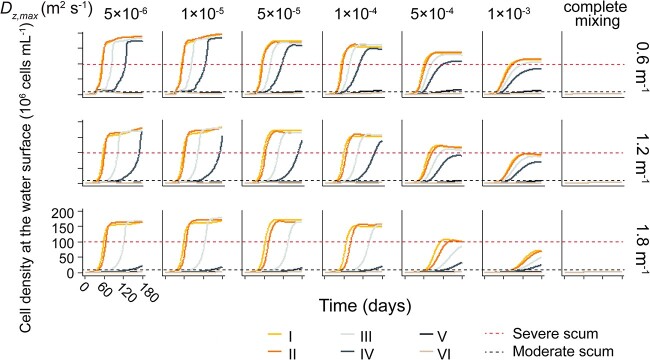
Time series of the cell density in the uppermost depth layer of *Microcystis* populations with different spectra of *P_max_* (population I to IV, see legend) under different turbulence (columns) and turbidity (characterized by *K_bg_* in m^−1^). The red dashed horizontal lines show the threshold for surface scum.

We found that low turbulence promoted the growth of population with wide ranges of *P_max_*, while high turbulence promoted the growth of population with more narrow ranges of *P_max_* (Fig. 4). This resulted in differences of total cell density between different populations with different ranges of *P_max_* decreased with increasing turbulence. Under complete mixing conditions, the total biomass of populations with different ranges of *P_max_* showed similar levels (Fig. 4). Under moderate and low turbulence conditions, the initially more diverse population (with initially wider range of *P_max_*) exhibited profoundly higher cell densities compared to the populations with a narrower range. The cell density of population I was found to be up to 2.5 times higher than that of population VI under such conditions (Fig. 4). In contrast, high turbidity tended to increase the difference of total cell density among different populations and decreased the maximum cell density. Similarly, our results showed that *Microcystis* populations with a wider initial range of *P_max_* can form denser scum layers. We observed higher cell density in the surface scum layer for populations with a wide range of in *P_max_* over the period of 180 days, particularly in populations I, II, and III (Fig. 5).

**Figure 6:**
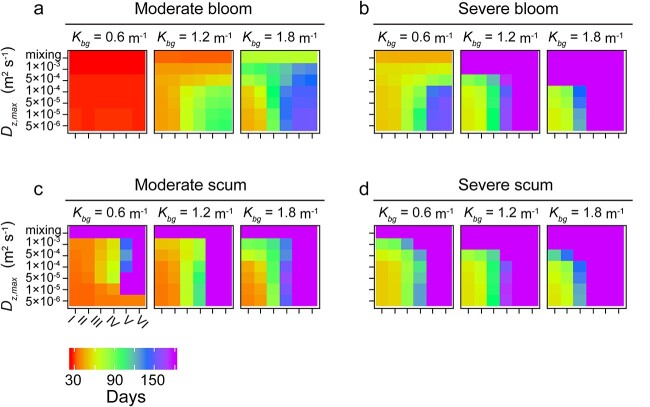
Time required for *Microcystis* populations with varying initial ranges of *P_max_* (population I-VI) to form blooms (moderate bloom, panel A and severe blooms B) and surface scum (moderate scum, panel C and severe scum, D) under different turbulence (rows) and turbidity (columns) conditions. Time is indicated by color, according to the color bar below the graphs.

The timescale for moderate and severe bloom formation ranged from 26 to >180 days. Similarly, we observed that the duration for the *Microcystis* population to develop blooms generally decreased as the range of intraspecific variations in *P_max_* expanded (Fig. 6). However, under *K_bg_* = 0.6 m^−1^, the time required for all the tested populations to form a moderate bloom was similar, ranging from 26 to 27 days (Fig. 6A). This suggests that the influence of turbulence and intraspecific variation in photosynthetic capacity on the occurrence of moderate blooms is not apparent at low turbidity, likely due to the comparable growth rates among the different populations in well-illuminated environments. Generally, a wide range of *P_max_*, low turbidity and low turbulence were accelerating bloom formation (Fig. 6A). The effect of turbidity on the formation of blooms and scum depended on the intensity of turbulence. Under low turbulence (diffusivities <1 × 10^−4^ m^2^ s^−1^), the total cell density was marginally affected by turbidity, while under higher turbulence (diffusivities >1 × 10^−4^ m^2^ s^−1^), the total cell density under *K_bg_* of 1.8 m^−1^ decreased by up to 55%, compared to that under *K_bg_* of 0.6 m^−1^ (Fig. 4). This result is likely because *Microcystis* tended to float to the water surface under conditions of low turbulence, which minimizes the impact of turbidity.

The formation time of surface scum was negatively correlated to the range of *P_max_* within the tested population (Fig. 6C and D). Population I showed a significant reduction in the timescale required to form moderate surface scum, up to more than four times faster than population VI (Fig. 6C). By contrast, populations with an initially narrow range of *P_max_* (e.g. Population IV, V, and VI) were unable to develop severe blooms or scum during the simulation period (Fig. 6). Surface scum was also less likely to occur under high turbidities (1.8 m^−1^) and high turbulences (diffusivity of 5 × 10^−4^ and 1 × 10^−3^ m^2^ s^−1^, and mixing conditions, Fig. 6C and D).

## Discussion

### Benefits of diversification of photosynthetic capacity for *Microcystis* populations

In this study, we investigated the selective process for the photosynthetic capacity of *Microcystis* traits under various simplified mixing dynamics and turbidity conditions typical for eutrophic and shallow lakes. Our findings demonstrate that both turbulence and turbidity play important roles in reshaping the population composition and the population-averaged photosynthetic capacity. Turbulence can selectively shift the population-averaged photosynthetic capacity towards either a high or low photosynthetic rate, primarily depending on the intensity of turbulence (Fig. 2). High turbidity, in contrast, accelerates the change in population-averaged photosynthetic capacity, likely by increasing light limitation and the competition for light. It also affects the trait composition and population dynamics of *Microcystis* (Fig. 3).

The underlying basis of the plasticity in population-averaged photosynthetic capacity lies in the contrary role of photosynthetic capacities in the migration and growth of *Microcystis*. *Microcystis* with high photosynthetic capacity have a greater growth potential but readily accumulate excessive ballast, which hinders access to light in the upper layers. Conversely, *Microcystis* with low photosynthetic capacity exhibit higher flotation velocities (Fig. S7, see online supplementary material for a colour version of this figure), and consequently tend to position at shallower depths to capture additional light. Our results show that fast-floating *Microcystis* (e.g. trait group g_1_) can stratify, if the turbulent diffusivity is <10^−3^ m^2^ s^−1^ (Fig. S6B, see online supplementary material for a colour version of this figure), similar to findings in previous studies [33, 42]. By the intraspecific diversification of the photosynthetic capacity, the *Microcystis* population can accumulate traits with the fittest photosynthetic capacity (accompanied by changes in population composition) across variable environmental conditions, optimizing the utilization of light resources for growth. The flexibility observed in the photosynthetic capacity of cyanobacterial populations, brought to light by our simulations, offers insights into how turbulence and turbidity might affect the population dynamics and primary production.

We also simulated the development of bloom and scum by *Microcystis* population with different initial diversities (range) of photosynthetic capacity. The simulated maximum depth-averaged cell density of *Microcystis* (~3.2 × 10^6^ cells mL^−1^), and cell density of surface scum (~1.8 × 10^8^ cells mL^−1^) under ideal conditions exceed those observed during bloom periods in lake Taihu and lake Dianchi, China [46, 47], but are comparable to the values observed in Nakdong River (South Keara), Central Park Lake, and Prospect Park Lake (USA) [48, 49]. Depending on the diversity of photosynthetic capacity and environmental conditions, the timescale for surface bloom formation varied from 26 to 180 days, which is consistent with observations in natural systems during cyanobacterial bloom periods [48–51]. Our results reproduce the diel migration pattern of *Microcystis* (Fig. S3) that is commonly observed in lakes [20], supporting the validity of the model. The main simulation results also showed good consistency with the supplementary simulations applying different initial conditions, indicating that the model is not very sensitive to changes in the initial vertical distribution and colony size.

Our results further demonstrate that high diversity of photosynthetic traits within the seed population promotes bloom formation of *Microcystis* populations across a broad range of physical environmental conditions, particularly under conditions of high turbidity and low turbulence (Fig. 4). This finding can be attributed to the fact that high diversity provides a larger pool of traits for selection to act upon and ensures a more efficient use of available light. Because of the adaptations, the diversity of photosynthetic capacity also plays an important role in the formation of scum layers. The diversity of photosynthetic capacity largely determines whether scum can occur within a 180-day period in shallow eutrophic lakes (Fig. 6). A wide range of photosynthetic capacity accelerate the formation of dense scum layer by decreasing the timescale for its development by up to > 4-fold, compared to more narrow ranges (Fig. 6).

The general paradigm suggesting that higher intraspecific trait diversity facilitates faster population growth has been proposed in prior studies [8, 52, 53], but its specific effects on cyanobacteria and surface scum has not been explored. The findings presented here indicate that this paradigm is applicable to cyanobacteria. As such, our study introduces a new perspective for investigating and predicting cyanobacterial blooms.

### Environmental relevance and limitations

Although the model is limited to a narrow range of environmental conditions, i.e., period of relatively stable water condition with no significant nutrients limitation, our results have clearly highlighted the importance of the photosynthetic trait in shaping the *Microcystis* population dynamics and could offer an alternative explanation for some field observations. For example, in Lake Kasumigaura, despite a relatively stable mean water temperature during the two periods in July to August and September to October, there was a significant decrease in *P_max_* of *Microcystis* during the first period, followed by an increase during the second period [27].

We expect that the interactions observed between cyanobacteria with diverse traits, turbulence, and light in our study would similarly apply to more dynamic conditions and exert varying degrees of influence on the photosynthetic capacity and population dynamics within dynamically changing real-world environments. To enhance the understanding of *Microcystis* dynamics under more dynamic environmental conditions, it would be advantageous to incorporate intraspecific trait variations into more complex models, such as integrating additional ecological factors (e.g. nutrient limitation) and utilizing more sophisticated hydrodynamic models such as including variable atmospheric forcing [21]. It is worth noting that obtaining the initial conditions of trait composition, which are necessary and essential for the trait-based model, could be achieved by collecting a substantial number of samples from lakes.

Our model did not account for all the processes that may directly or indirectly influence the dynamics of cyanobacterial populations due to the partially unclear mechanisms involved. For example, the plastic photosynthetic capacity response of traits to the environment (e.g. temperature and nutrient); the additional interactions among *Microcystis* colonies; and the intricate interplay between colony size and the environment, which also involves colony disaggregation and cell adhesion [39, 40, 54]. Particularly, cell adhesion may facilitate the formation of multi-trait colonies. Furthermore, our study only considered variations in one key trait, whereas additional traits, such as toxigenicity and the optimal light intensity of the P-I curve, may also influence population dynamics. The latter may allow certain groups to reach different *P_max_* at lower or higher irradiance than other groups within each population. With such differentiation, populations that might more effectively exploit a wider range of available irradiance, i.e. population with less niche overlap, are expected to outperform the type of population currently utilized in our study, at least under some of our modelled environmental conditions. A more accurate depiction of the intricate population dynamics would include these processes and more traits variations.

Our finding of a consistently dominant population characterized by low photosynthetic capacity (Fig. 2) under periodic moderate turbulence presents an intriguing possibility to employ artificial mixing techniques for manipulating traits and their diversity within the *Microcystis* population. For example, implementing controlled, moderate artificial mixing during bloom events could potentially reduce the diversity of photosynthetic capacity, lower the resilience of the population, and mitigate future blooms. This concept offers a new theoretical foundation for using artificial mixing as a means to mitigate blooms [55].

Recently, increasing underwater light attenuation due to ongoing anthropogenic pollution has been observed [56]. The increased turbidity is expected to enhance buoyancy [36] but concurrently reduces the growth of *Microcystis*. Although our model predicts an overall negative impact of turbidity on scum formation (Figs 4 and 5), our results also revealed an intensification of differences in bloom formation among populations with varying diversities due to elevated turbidity (Fig. 4). Hence, we call for the inclusion of intraspecific variation to better predict how physical environmental variables affect ecosystem productivity and shape the fate of cyanobacteria in present-day and future environmental conditions.

## Conflicts of interest

The authors declare no competing financial interests.

## Funding

This study was financially supported by the German Research Foundation (grant no. LO 1150/18) and the National Natural Science Foundation of China (grant no. 42061134013).

## Data availability

The scripts and datasets in the current study are available at the Knowledge Network for Biocomplexity (KNB) repository (doi:10.5063/F1V986J0).

## Supplementary Material

SupportingInformation_wrae021
