# Supplementary material for: Selection of photosynthetic traits by turbulent mixing governs formation of cyanobacterial blooms in shallow eutrophic lakes
Source: ISME J. 2024 Feb 2;18(1):wrae021. doi: 10.1093/ismejo/wrae021 (PMC10945370; doi:10.1093/ismejo/wrae021)
Supplement: SupportingInformation_wrae021 [file supportinginformation_wrae021.docx]

# Supporting Information for:

# Selection of photosynthetic traits by turbulent mixing governs formation of cyanobacterial blooms in shallow eutrophic lakes

Huaming Wu^a*^, Xingqiang Wu^b^, Lorenzo Rovelli^a,c^, Andreas Lorke^a^

*^a^Institute for Environmental Sciences, University of Kaiserslautern-Landau (RPTU), Landau 76829, Germany*

*^b^Key Laboratory of Algal Biology of Chinese Academy of Sciences, Institute of Hydrobiology, Chinese Academy of Sciences, Wuhan 430072, China*

*^C^now at the Department of Ecology, Federal Institute of Hydrology (BfG), Koblenz 56068, Germany*

*Corresponding author.

Tel.: +49 06341-28031824

E-mail address: wu.h@rptu.de

**Text: Methods**

#### Text S1

The photosynthetic capacity of phytoplankton can be influenced by other environmental conditions, including temperature, nutrients, and pollutants [1-3], however, there is currently no universally established relationship between photosynthetic capacity and these factors. Moreover, the alteration of population-averaged *P_max_* is driven by intraspecific variations and competition, both of which are factors not expected to be eliminated. To isolate and investigate the specific effects of turbulence and turbidity on the population-average *P_max_*, we excluded the impacts of other environmental conditions in this study and assumed a fixed *P_max_* for each trait group.

#### Text S2: Colony size and migration velocity

The rising/sinking velocity of colonies was derived as a function of colony size *d* and cell tissue density *ρ* using Stokes' law:

$\frac{\partial z}{\partial t}\approx\frac{gd^{2}(\rho_{col-}\rho_{w})}{18\mu\varphi}$ (S1)

where *g* (m s^-2^) is gravitational acceleration, *ρ_w_* (kg m^-3^) is the density of water, *ρ_col_* (kg m^-3^) is the mass-density of colonies, which is estimated from the cell tissue density (see below), *φ* is a shape coefficient and *µ* is the dynamic viscosity of water (kg s m^-2^). According to Stokes’s law, colonies with larger size have higher floating velocities.

We estimated the mass-density of the colony (*ρ_col_*) as follows:

$\rho_{col}=\rho\cdot(1-n_{gas})\cdot n_{cell}+\rho_{mul}\cdot(1-n_{cell})$ (S2)

Where *ρ* is the cell tissue density (cf. Eq. 4 in the main text), *n_gas_* is the volumetric ratio of gas vesicles to the total cell, *n_cell_* is the volumetric ratio of cells to the colony. *ρ_mul_* is the mass-density of mucilage. The values used here are shown in Table S1.

#### Text S3: External environmental conditions

We assumed that the vertical dispersion of colonies can be described by the turbulent diffusivity *D_z_*, given that their size was smaller than the Kolmogorov micro scale of turbulence by over one order of magnitude, and assuming that vertical mixing was independent of cell-tissue density and colony size, i.e., we neglected the diffusion term in the *ρ* and *d* dimensions in Eq. 1 [4].

The turbulent diffusivities at depth *z* (*D_z_*) were estimated following [5]:

$D_{z}=\left\{ \begin{aligned} a\left[ z-\frac{z^{2}}{H} \right], z\geq\frac{H}{10} \\ \\ a\frac{H}{10}, z<\frac{H}{10} \end{aligned} \right.$ (S3)

Where *a* (m s^-1^) is a constant, and *H* is the maximum water depth. The maximum turbulent diffusivity (*D_z,max_*) derived from eq. S2 is *a*×*H*/4. Here we varied the value of *a* (6.67 × 10^-6^ – 1.33 × 10^-3^ m s^-1^) to calculate the turbulent diffusivity profiles with different *D_z,max_*.

The irradiance profiles were simulated by Lambert–Beer’s law, taking into account the self-shading of the *Microcystis* population and a constant background turbidity that was varied in our simulations:

$I(z,t)=I_{surf}e^{\{-\int_{0}^{z} \left[ K_{m}\sum_{i} C_{i}\left( \delta,t \right) \right]d\delta-K_{bg}z\}}$ (S4)

Where *I(z,t)* is the light intensity at depth *z* and time *t*, *I_surf_* is the light intensity at the water surface, which was simulated by a sinusoidal pattern during daytime (*I_surf_*=*I_max_*×sin(πt/L) from 6:00 – 18:00 and set to zero at night (18:00 – 06:00). *I_max_* is the maximum daily light intensity and is set to 1000 µmol photon m^-2^ s^-1^. *L* is the duration of daytime and is set to 12 h. *K_m_* (0.0084 cm^2^ 10^‒6^ cells^‒1^) [6] and *K_bg_* (0.6 – 1.8 m^-1^, [6, 7]) are the extinction coefficients of *Microcystis* and algae-free water (background), respectively. Σ*C_i_*(*δ*,*t*) is the total cell density of all trait groups at time *t* and depth *δ*.

**Text S4: Cell density threshold for moderate and severe bloom and scum**

The depth-averaged cell density at 6:00 was used as a measure for bloom formation, while the cell density in the uppermost layer (~5 cm) served as an indicator for surface scum. A mean cell density of 0.5×10^6^ cells mL^-1^ was designated as the threshold for moderate blooms, while a threshold of 2×10^6^ cells mL^-1^ was used as a threshold for severe blooms. In the case of surface scum, a cell density of 1×10^7^ cells mL^-1^ at the uppermost layer indicated middle scum, suggesting a moderate accumulation of *Microcystis* at the water surface. Conversely, a cell density of 1×10^8^ cells mL^-1^ at the uppermost layer was considered severe scum, indicating a substantial and pronounced accumulation of *Microcystis* at the water surface.

Table S1: Definition, description, values, and references of the parameters used in the model.

| **Symbol** | **Description (unit)** | **Value** | **Reference** | |
| --- | --- | --- | --- | --- |
| *I_opt_* | Optimal light intensity (μmol photons m^-2^ s^-1^) | 277.50 | | [7] |
| *S* | Initial slope of P-I curve (h μmol photons m^-2^ s^-1^)^-1^) | 2.00 × 10^-7^ | | [8] |
| *g_max_* | Maximum carbon requirement for growth (s^-1^) | 5.50 × 10^-6^ | | [9] |
| *R* | Respiration rate (s^-1^) | 0.55 × 10^-6^ | | [9] |
| *V_cell_* | Volume a single *Microcystis* cell (m^3^) | 67.00 × 10^-18^ | | [9] |
| *l* | Loss rate of *Microcystis* (d^-1^) | 0.10 | | [10] |
| *g* | Gravitational acceleration (m s^-2^) | 9.80 | | [4] |
| *ρ_w_* | Density of water (kg m^-3^) | 998.00 | | [4] |
| *ρ_mul_* | Density of mucilage (kg m^-3^) | 998.70 | | [11] |
| *φ* | Shape coefficient of colonies | 1.00 | | [4] |
| *µ* | dynamic viscosity of water (kg m^-1^ s^-1^) | 1.00 × 10^-3^ | | [4] |
| *I_max_* | Maximum daily light intensity (μmol photons m^-2^ s^-1^) | 1000.00 | | [6] |
| *L* | Duration of daytime (h) | 12.00 | | [6] |
| *K_m_* | Extinction coefficient of *Microcystis* (cm^2^ 10^-6^ cells m) | 0.84× 10^-2^ | | [6] |
| *f_1_* | Slope of the curve of density change (min^-1^) | -9.49 × 10^-4^ | | [7] |
| *f_2_* | Theoretical rate of density change with no carbohydrate storage in the cells (kg m^-3^ min^-1^) | 9.84× 10^-1^ | | [7] |
| *m_cell_* | Amount of carbon contained in each cell (kg) | 14.00 × 10^-15^ | | [9] |
| *n_gas_* | volumetric ratio of gas vesicles to the total cell | 0.07 | | [6] |
| *n_cell_* | Volumetric ratio of cells to the colony | 0.20 | | [6] |
| *B_g_* | The mass of carbohydrate (glycogen) ballast produced per gram of assimilated carbon (g) | 2.38 | | [9] |
| *H* | The maximum water depth (m) | 3.00 | | This study |

**Figure legend：**


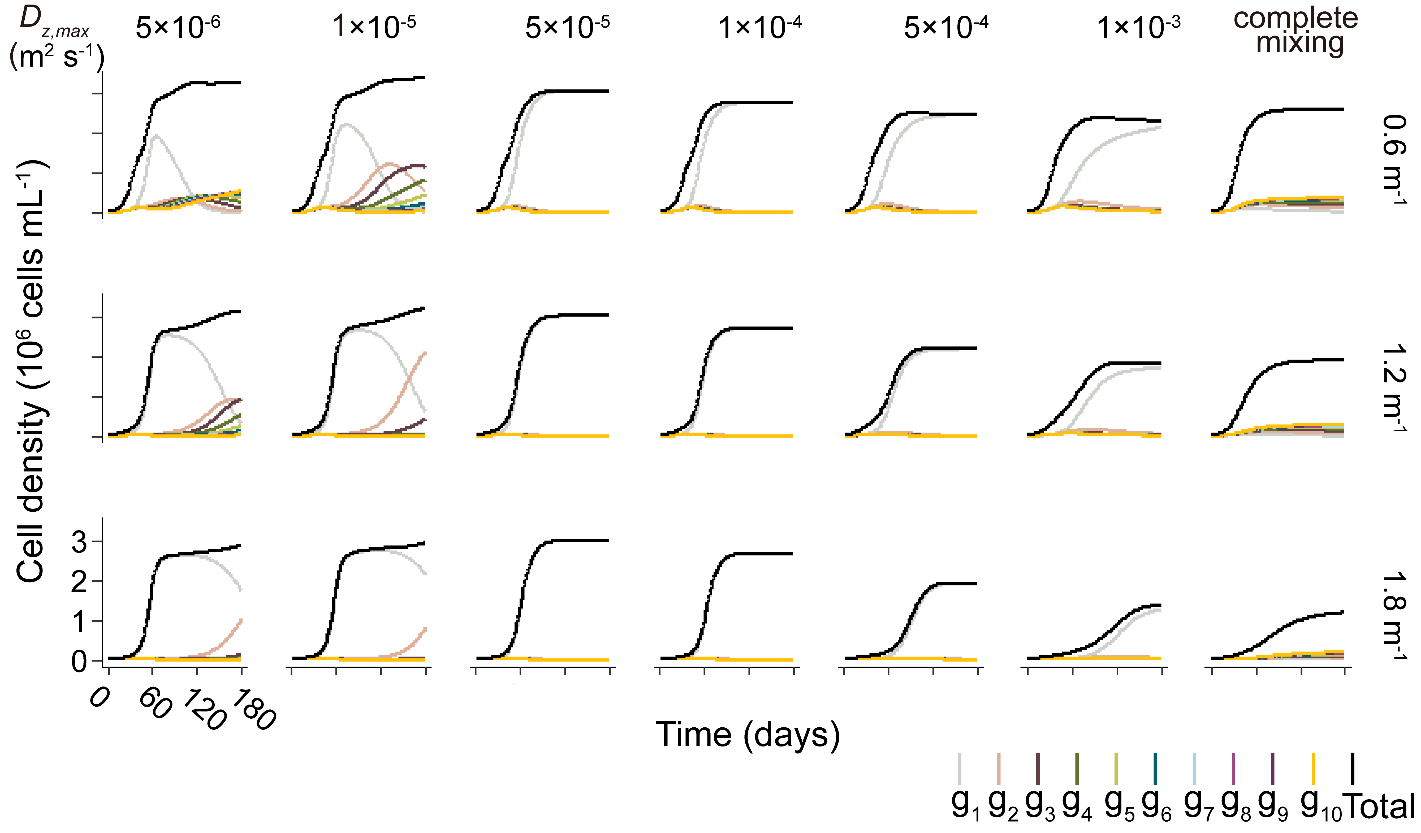


**Fig. S1:** Time series of cell density of the population (population I) and its trait groups (see color assignment) under different turbulent diffusivities (*D_z,max_*, see the columns) and turbidity (rows) conditions. Axis scaling is identical in all panels.


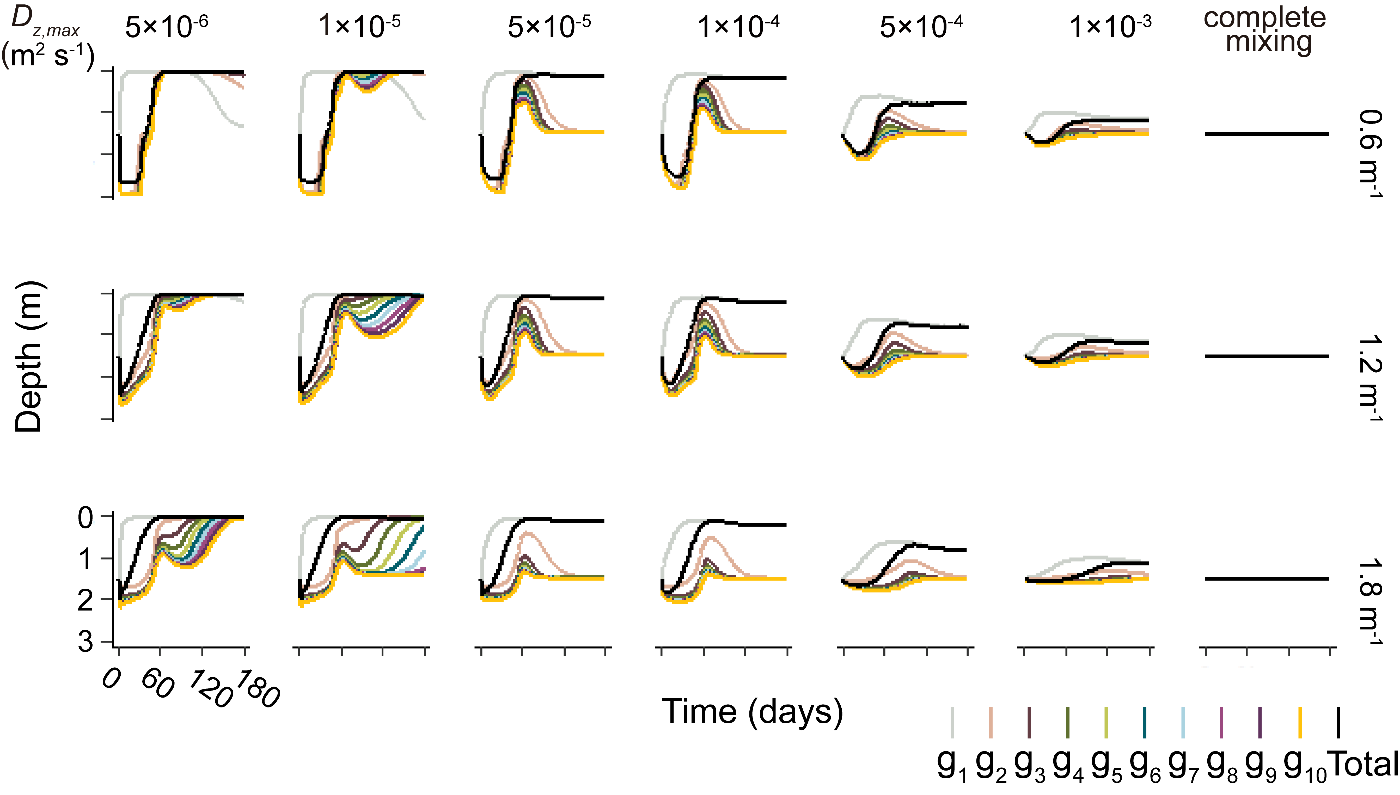


**Fig. S2:** Time series of the depth of the centroid of the population (population I) and traits groups (see color assignment) under different turbulence (columns) and turbidity (rows) conditions.


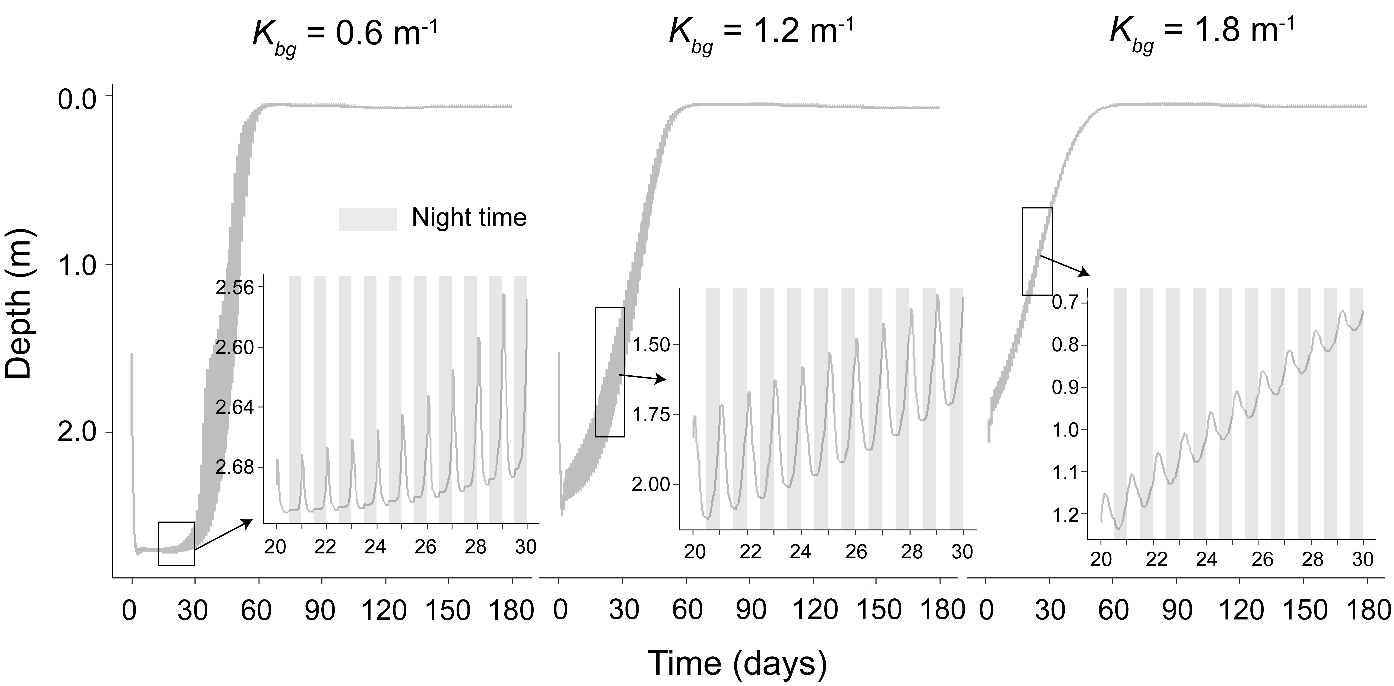


**Fig. S3**: Hourly time series of the centroid depth of Population I under different turbidity conditions (columns), exemplified for weak turbulence (*D_z,max_* = 5×10^-6^ m^2^ s^-1^). The inset graphs highlight the diel vertical migration by showing the data marked by the black rectangle at higher resolution. In these graphs, the grey-shaded area mark night time conditions (no light).


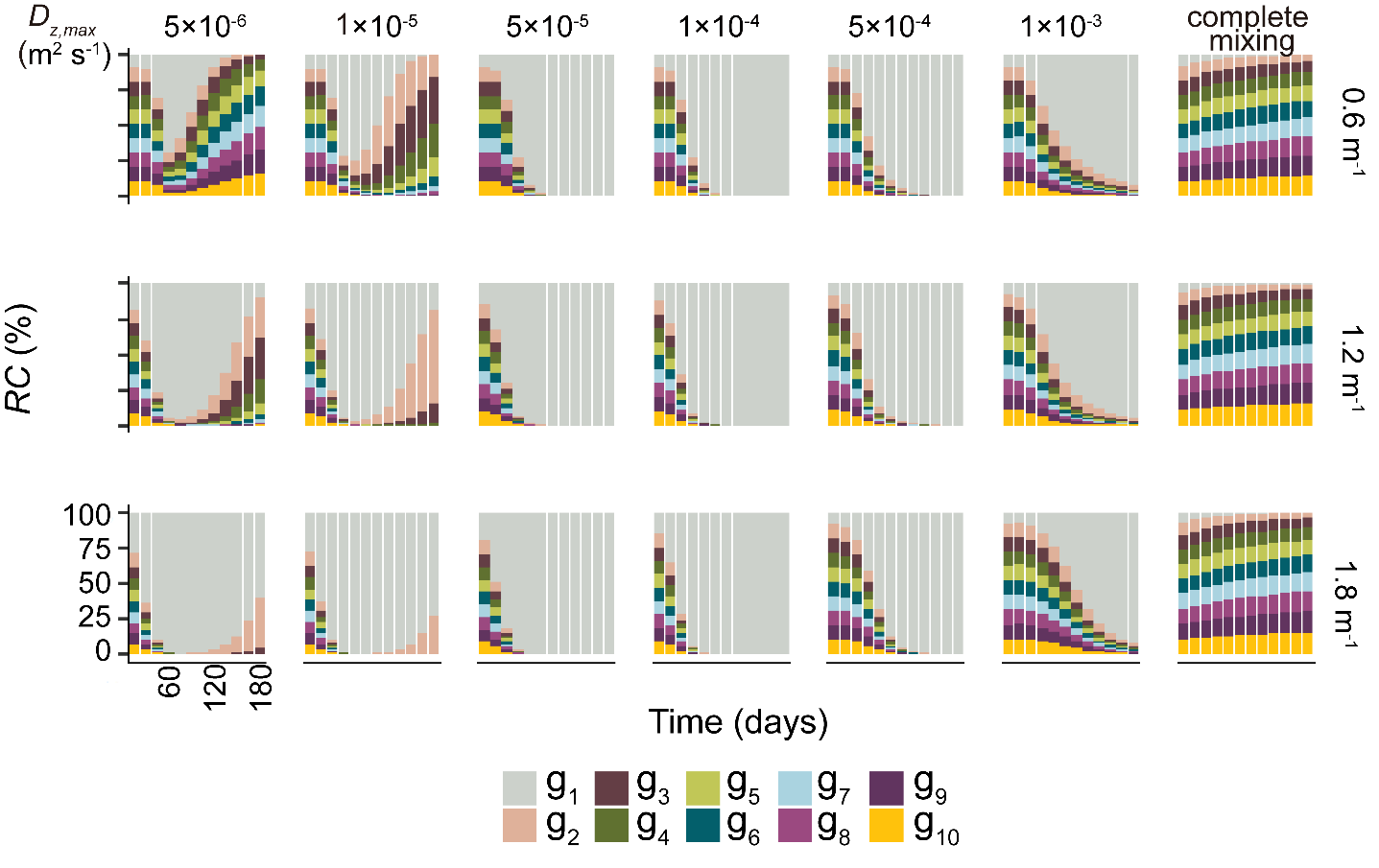


**Fig. S4:** Staggered bar charts showing time series of the relative cell density (*RC*) of the different trait groups (see color assignment, Population I) under different turbulence columns) and turbidity (rows) conditions.


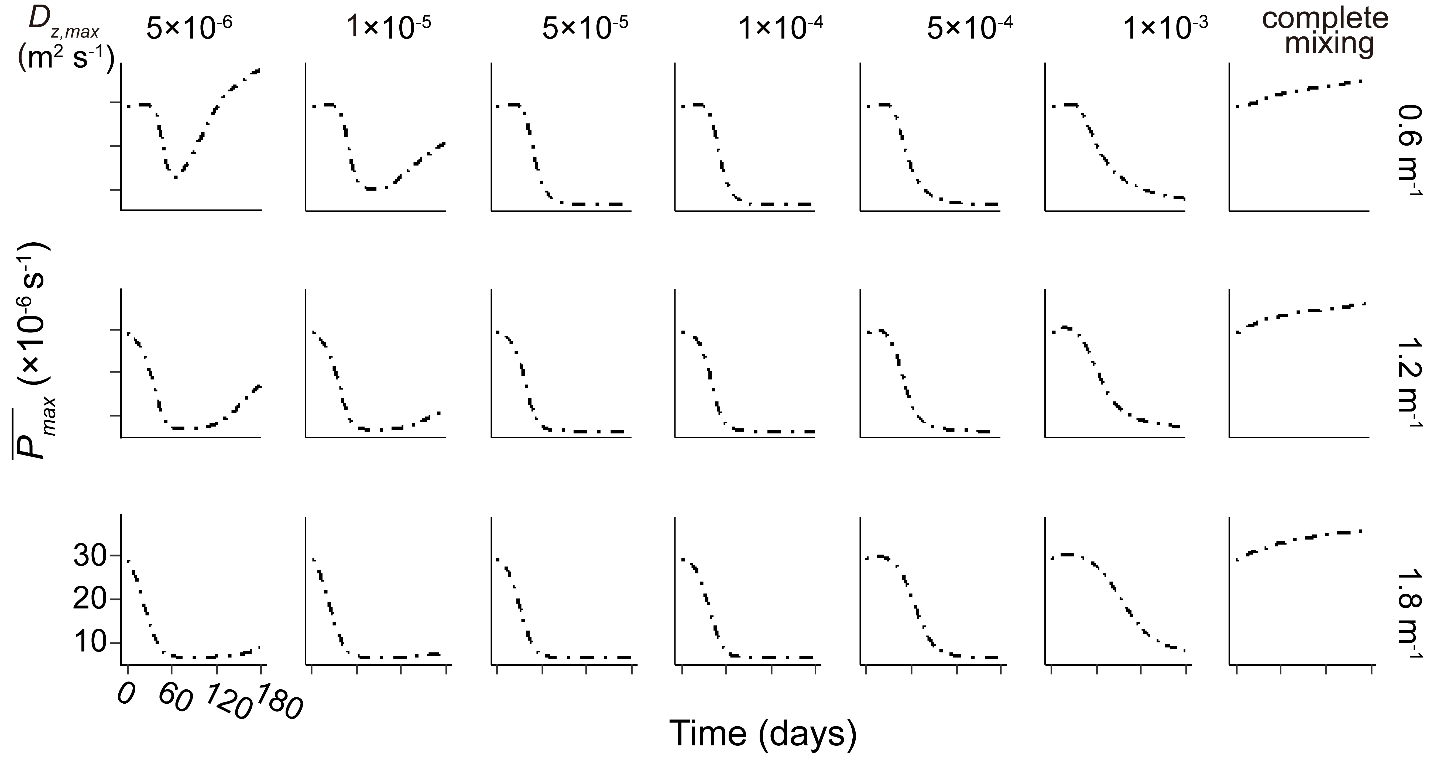


**Fig. S5:** Time series of population-averaged photosynthetic capacity (*P_max_*) of population I under different turbulence (columns) and turbidity (rows) conditions.


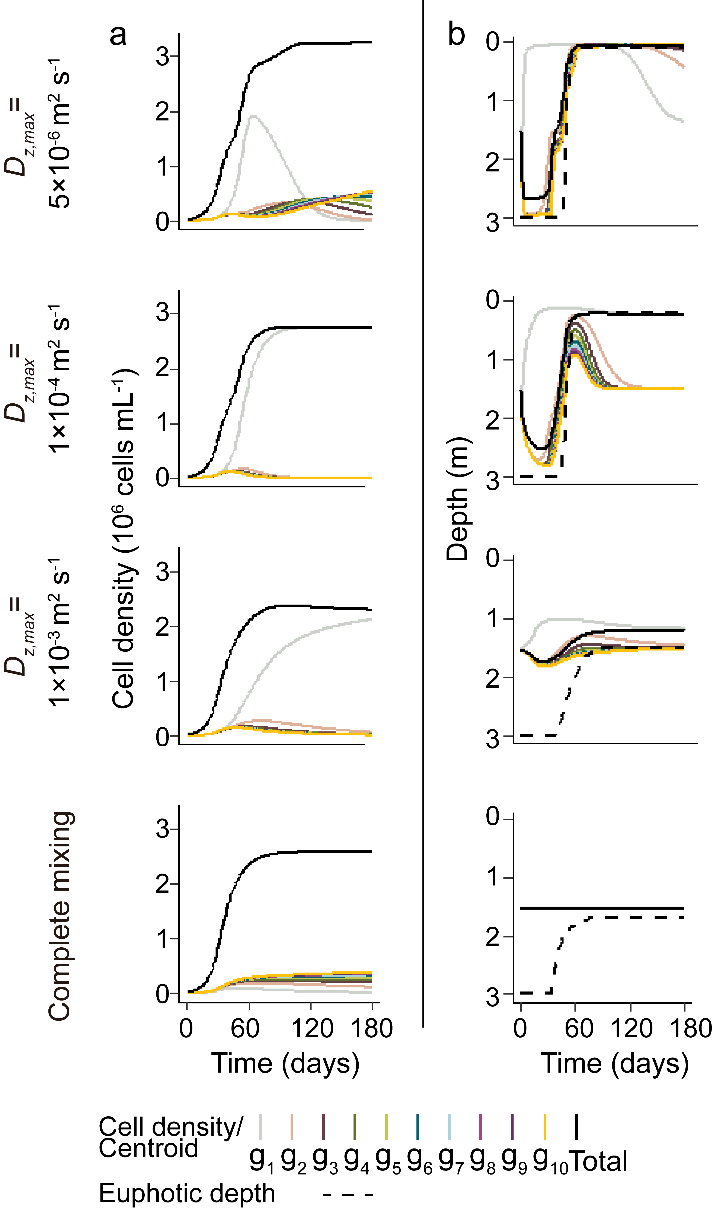


**Fig. S6**: **Population dynamics of *Microcystis* (population I) for four different turbulence conditions** (different rows: *D_z,max_*=5×10^-6^ m^2^ s^-1^; *D_z,max_* =1×10^-4^ m^2^ s^-1^; *D_z,max_* =1×10^-3^ m^2^ s^-1^; complete mixing). Different columns show time series of cell density (**a**) and depth of the centroid (**b**) of the 10 different trait groups (g_1_ – g_10_, see the color assignments). Group g_1_ has the minimum *P_max_*, while g_10_ has the maximum *P_max_*. The black solid lines in the panel **a** and **b** represent the cell density and the centroid of the overall *Microcystis* population, respectively. The black dashed line represents the euphotic depth. The turbidity conditions were *K_bg_*=0.6 m^-1^.

**
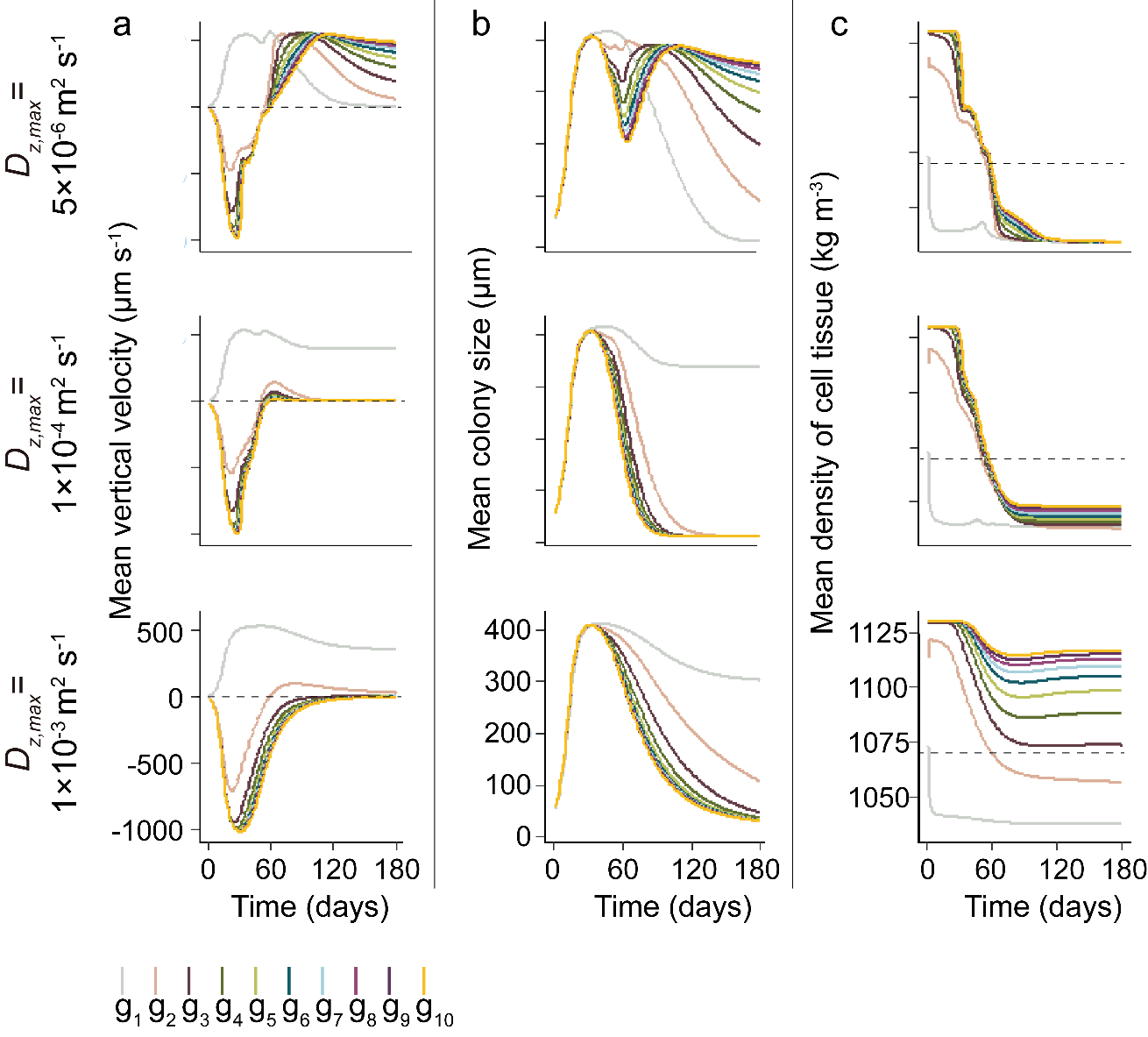
**

**Fig. S7:** Time series of vertical velocity (a), colony size (b) and density of cell tissue (c) of different trait groups (see color assignment) of population I under different turbulence conditions (different rows) and a background extinction coefficient (turbidity) of 0.6 m^-1^. The horizontal dashed lines in panel a indicate a vertical velocity of zero and the dashed lines in panel c represent neutral buoyancy of colonies.


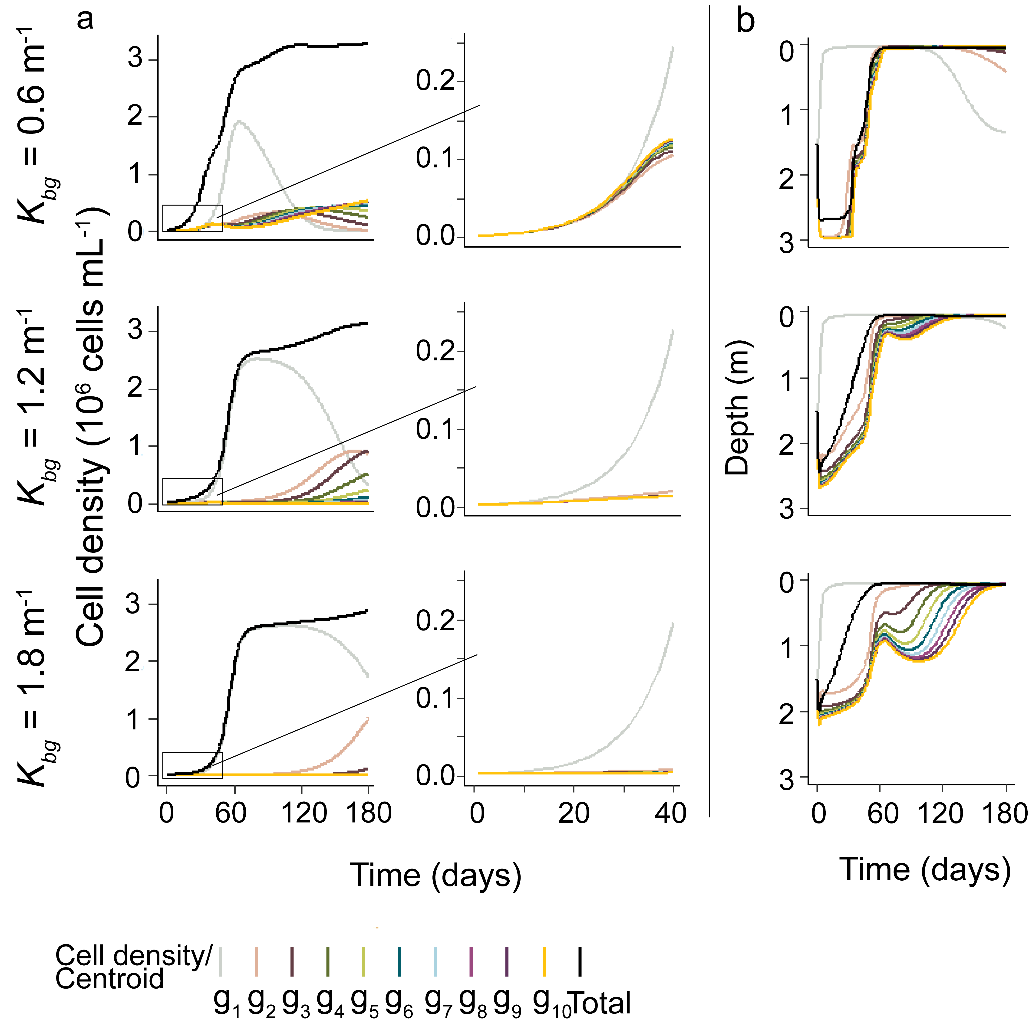


**Fig. S8: The effect of turbidity on the population dynamics of *Microcystis* (Population I) under weak turbulence (***D_z,max_* **= 5×10^-6^ m^2^ s^-1^**) (see different rows: *K_bg_*=0.6 m^-1^; *K_bg_* =1.2 m^-1^; *K_bg_* =1.8 m^-1^): Different columns show time series of cell density (**a**) and depth of the centroid (**b**) of the 10 different trait groups (g_1_ – g_10_, see the color assignments). Group g_1_ has the minimum *P_max_*, while g_10_ has maximum *P_max_*. The black solid lines in the panel **a** and **b** represent the cell density and the centroid of the overall *Microcystis* population, respectively.


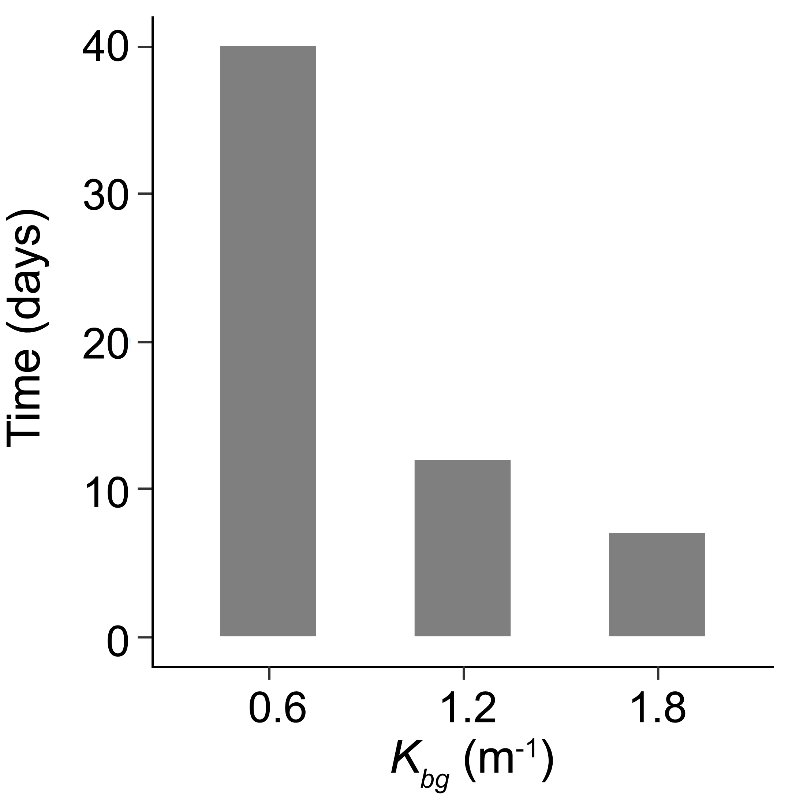


**Fig. S9**: The time at which the population-averaged photosynthetic capacity (*P_max_*) deviated more than 5% of its initial value for different turbidity conditions (background extinction coefficients *K_bg_*).


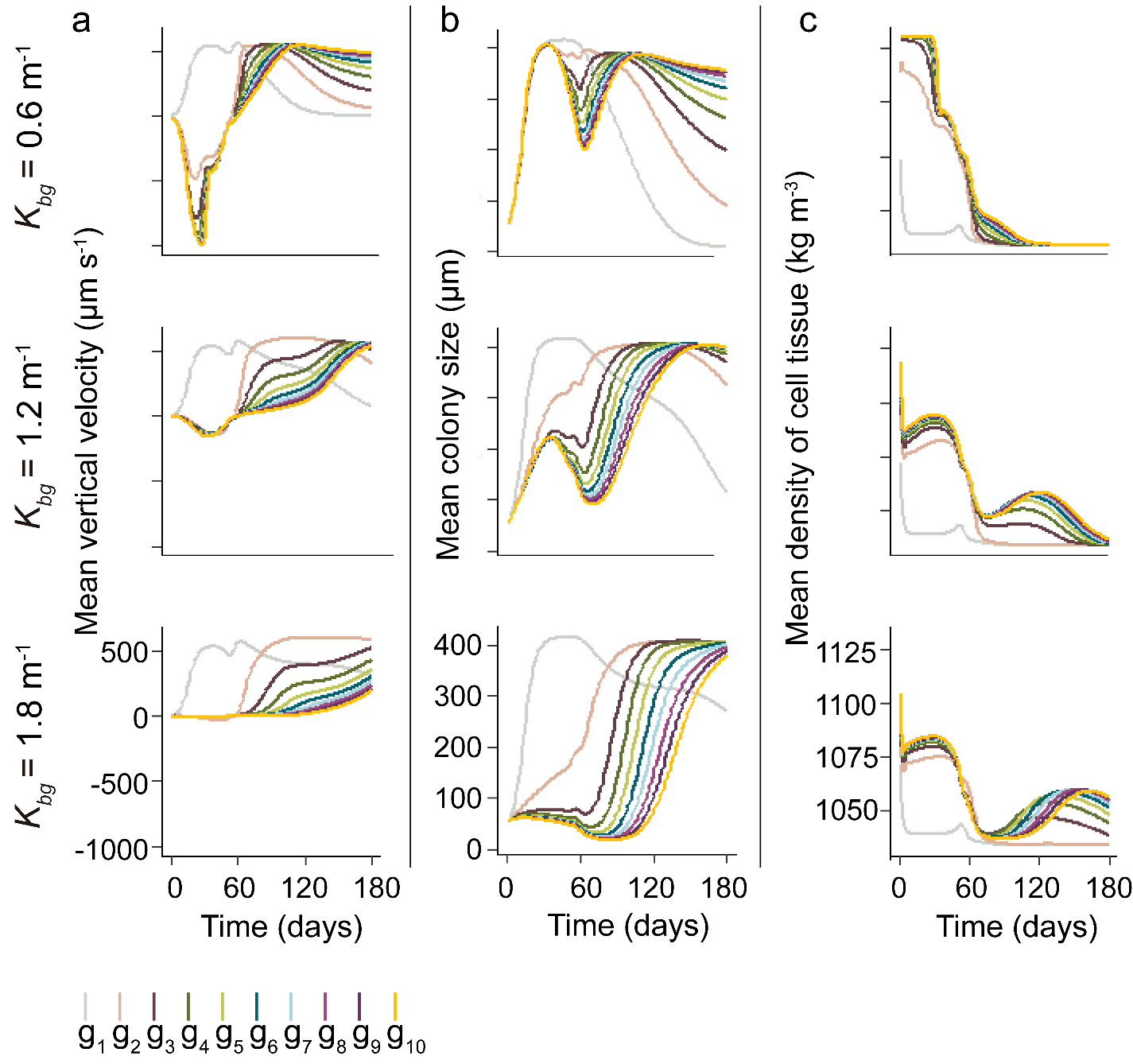


**Fig. S10:** Time series of vertical velocity (a), mean colony size (b) and density of cell tissue (c) of different trait groups (see color assignment below the graphs) of population I under different turbidity conditions (different rows). All results were obtained for a turbulent diffusivity of *D_z,max_* = 5 ×10^-6^ m^2^ s^-1^.


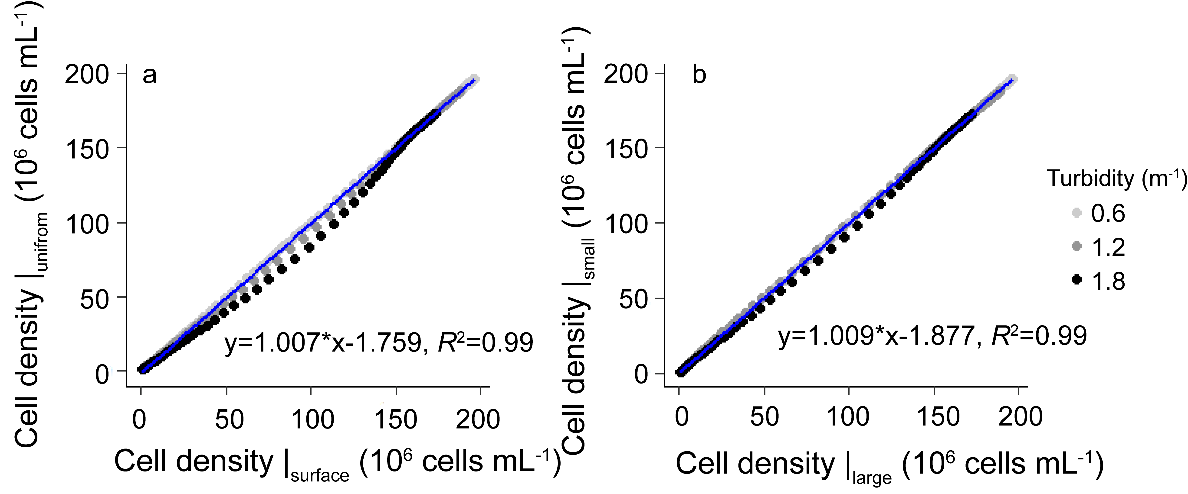


**Fig. S11:** Comparison of cell density simulated for different initial conditions: **a**) Initially uniform distribution (Cell density|_unifrom_) vs. an initial distribution where colonies were concentrated at the water surface (Cell density|_surface_), and **b**) initially large colony size (95 μm, Cell density|_large_) vs. initially small colony size (50 μm, Cell density|_small_). The simulations were performed under different turbidity conditions (see the color assignments) and under weak turbulence (*D_z,max_* = 5×10^-6^ m^2^ s^-1^).

**Reference**

1. Wang Z, Li D, Li G, Liu Y. Mechanism of photosynthetic response in *Microcystis* *aeruginosa* PCC7806 to low inorganic phosphorus. Harmful Algae 2010;9:613-619.

2. Zhou S, Shao Y, Gao N, Deng Y, Qiao J, Ou H, et al. Effects of different algaecides on the photosynthetic capacity, cell integrity and microcystin-LR release of *Microcystis* *aeruginosa*. Sci Total Environ. 2013;463-464:111-119.

3. Davison IR. Environmental effects on algal photosynthesis: temperature. J Phycol. 1991;27:2-8.

4. Aparicio Medrano E, Uittenbogaard RE, Dionisio Pires LM, van de Wiel BJH, Clercx HJH. Coupling hydrodynamics and buoyancy regulation in *Microcystis* aeruginosa for its vertical distribution in lakes. Ecol Model. 2013;248:41-56.

5. Webster. IT, Hutchinson PA. Effect of wind on the distribution of phytoplankton cells in lakes revisited. Limnol Oceanogr. 1994;39,:365-373.

6. Wu H, Wu X, Yang T, Wang C, Tian C, Xiao B, et al. Feedback regulation of surface scum formation and persistence by self-shading of *Microcystis* colonies: Numerical simulations and laboratory experiments. Water Res. 2021;194:116908.

7. Visser PM, Passarge J, Mur LR. Modelling vertical migration of the cyanobacterium Microcystis. Hydrobiologia 1997;349:99–109.

8. Takamura N, Iwakuma T, Yasuno M. Photosynthesis and primary production of *Microcystis aeruginosa* Kütz. in Lake Kasumigaura. J Plankton Res. 1985;7:303-312.

9. Reynolds CS. Temporal scales of variability in pelagic environments and the response of phytoplankton. Freshw Biol. 1990;23:25-53.

10. Huisman J, Sharples J, Stroom JM, Visser PM, Kardinaal WEA, Verspagen JMH, et al. Changes in turbulent mixing shift competition for light between phytoplankton species. Ecology 2004;85:2960-2970.

11. Reynolds C, Jaworski G, Cmiech HL, G. On the annual cycle of the blue-green alga *Microcystis* *aeruginosa* K¨utz. emend. Elenkin. Philos. Trans. R. Soc. Lond., B, Biol. Sci. 1981;293, 419–477.
